# Addressing concerns about sustainability and animal welfare: Investigating consumers' adoption and behavioral intention towards plant‐based eggs

**DOI:** 10.1002/fsn3.4398

**Published:** 2024-08-23

**Authors:** Han‐Shen Chen, Ching‐Tzu Chao, I‐Kai Lin

**Affiliations:** ^1^ Department of Health Industry Technology Management Chung Shan Medical University Taichung Taiwan; ^2^ Department of Medical Management Chung Shan Medical University Hospital Taichung Taiwan

**Keywords:** animal welfare, plant‐based egg, purchase intention, sustainability

## Abstract

This study aimed to address the concerns regarding sustainability and animal welfare that have led to egg shortages. By examining consumer adoption and purchase intention towards plant‐based eggs as a viable alternative, this study identifies the key factors influencing consumer interest. The study integrates the Theory of Planned Behavior (TPB) and Value‐Attitude‐Behavior (VAB) frameworks. Data from 239 responses were collected through a questionnaire survey and analyzed using SPSS 27.0 and AMOS 28.0. The results highlight the significant impact of intrinsic food value and sensory appeal on purchase intention. Attitude, subjective norms, and perceived behavioral control mediate the relationship between food value and purchase intention. The findings of this study provide valuable insights for food industry professionals by enabling them to develop targeted strategies. Moreover, this study emphasizes plant‐based eggs as a sustainable and health‐conscious solution to egg shortages, thereby promoting overall industry sustainability.

## Graphical abstract

This study combines the Theory of Planned Behavior (TPB) and Value‐Attitude‐Behavior (VAB) frameworks to show that the intrinsic value of food and its sensory appeal significantly affect consumers' willingness to purchase plant‐based eggs. These findings offer valuable insights for food industry professionals, enabling them to develop targeted strategies that capitalize on the appeal of plant‐based eggs as both eco‐friendly and health‐conscious choices.

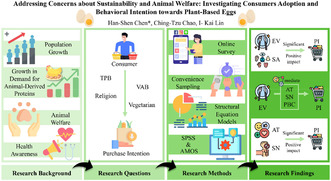

## 1 INTRODUCTION

The rapid expansion of the global population, together with the rising demand for animal proteins, poses a significant challenge for many existing food production systems. These systems are now considered unsustainable because of their harmful effects on the environment, as described by Godfray et al. (2010) and Godfray (2019). This situation has encouraged us to rethink traditional farming methods and sparked global interest in developing sustainable alternatives that are considerate towards animal welfare and consumer health consciousness (Short et al., 2021).

Data from the Food and Agriculture Organization (FAO) (2022) show a steep rise in worldwide egg production, reaching 87 million tons in 2020 and a jump of 36 million tons since 2000. This emphasizes humanity's reliance on animal proteins, which further complicates the issue, as livestock farming significantly increases greenhouse gas emissions. This necessitates urgent exploration of alternative food production methods (Clark et al., 2020).

Plant‐based products have emerged as possible substitutes in this critical situation. These products use plant materials to mimic the properties of animal‐based foods, providing practical choices with reduced environmental impact. Furthermore, these plant‐based innovations have attracted a growing health‐conscious market motivated by concerns about animal welfare and environmental sustainability (Aschemann‐Witzel et al., 2021; Clark & Bogdan, 2019; Schiano et al., 2020; Szenderák et al., 2022; Valencia‐Flores et al., 2013).

Plant‐based eggs are key innovations in this context. They replicate the sensory attributes of animal eggs by using plants as crucial ingredients. They have incorporated stringent safety standards by avoiding concerns related to antibiotics and *Salmonella*, which are often found in traditional eggs. These also sidestep the significant financial and environmental costs associated with chicken farming (Taiwan Institute for Sustainable Energy, 2023; Whiley & Ross, 2015).

Plant‐based eggs offer major health benefits, as highlighted by the reduction in heart disease risk related to lowering cholesterol intake from chicken eggs (Zhong et al., 2019; Zhuang et al., 2021). This aligns with the current health concerns. Consequently, this shift towards plant‐based eggs could help alleviate the overuse of resources caused by constant consumer demand, especially considering recent issues such as bird flu outbreaks, changing consumer demands, and supply chain disruptions due to COVID‐19, which exacerbated the global egg shortage problem (Gunjan et al., 2023). If consumers decrease their chicken egg consumption and steadily shift to plant‐based eggs, this could effectively address environmental, health, and egg shortage issues. However, because of the high cost and insufficient marketing of plant‐based eggs, isolating factors that attract consumers to buy plant‐based eggs is vital, thereby providing an impetus for this study.

Consumer behavior research highlights the role of motivation in decision‐making (Houben et al., 2010). Past literature shows that food‐choice motivations shape attitudes and behaviors towards food. Consumer eating values are commonly used to explore specific food areas. Factors related to individual consumer attitudes and values, particularly those concerning animal welfare, significantly affect the choice and consumption of plant‐based products (de Graaf et al., 2016; Haas et al., 2019; McCarthy et al., 2017).

Rombach et al. (2023) stress that a product's green image is highly valued by environmentally conscious consumers. Furthermore, concerns about consuming a healthy diet free of animal fats significantly drive the selection and consumption of plant‐based products (Bus & Worsley, 2003a, 2003b; de Graaf et al., 2016; He et al., 2020; McCarthy et al., 2017; Vainio et al., 2016).

The Theory of Planned Behavior (TPB) is often used to analyze food preferences across product categories. Consumers' buying intentions are considerably influenced by attitudes, subjective norms, and perceived behavioral control (Bae & Choi, 2021; Budhathoki & Pandey, 2021; Carfora et al., 2019; Chang et al., 2019; D'Souza, 2022; Shen & Chen, 2020; Wong et al., 2018). A study by Pandey et al. (2021), applying the TPB, confirmed that positive attitudes, perceived behavioral control, and perceived sensory attributes resulted in a stronger intention to consume plant‐based yogurts.

Concurrently, researchers have also studied the role of customer value in shaping attitudes towards specific products and subsequently influencing purchase behavior, leading to the development of the value‐attitude‐behavior (VAB) model (Homer & Kahle, 1988). This model has been applied in various contexts to understand consumer behavior.

Many studies have combined the TPB with the VAB model to explore various topics, including hotel accommodation (Tajeddini et al., 2021), green cars (Wang et al., 2022), intangible cultural heritage (Li & Romainoor, 2024), and participation in green behaviors (Anuar et al., 2021). Therefore, integrating these two theories can provide a more comprehensive explanation of consumer behavior and enhance predictive accuracy.

Research has consistently indicated a complex interplay involving health consciousness, environmental awareness, and consumer behavior, particularly towards accepting plant‐based products (de Graaf et al., 2016; Haas et al., 2019; McCarthy et al., 2017). Similarly, their sensory appeal and individual attitudes towards new food types can either facilitate or hinder their acceptance (Baker et al., 2015; Piqueras‐Fiszman & Spence, 2015; Prescott et al., 2002). Despite these advancements, a complete understanding of what encourages consumers toward plant‐based alternatives remains elusive. This study aimed to address these gaps by examining the interplay of these determinants in the context of plant‐based egg consumption, adopting the TPB and the VAB model as the framework for analysis.

Research conducted by Milichovský and Mráček (2020) suggests that vegetarians adhere to dietary routines guided by moral principles, animal welfare, and ecological concerns. Consequently, this investigation considers these dietary habits when examining whether purchasing intentions for plant‐based eggs fluctuate due to such practices. Religious convictions have a noteworthy positive influence on attitudes towards eco‐friendly purchases, subjective norms, perceived behavioral control, and sustainable purchase intentions (Wang & Wong, 2020). As underscored by Alotaibi and Abbas (2023), Islamic religious beliefs considerably impact green food‐purchasing intentions among millennials. Hence, this study focuses on the subject's religious affiliations when analyzing whether the buying intentions for plant‐based eggs change because of religious beliefs.

A detailed exploration by Jaeger and Giacalone (2021) on impediments to consuming plant‐based beverages and edible snacks identified emotional, conceptual, and situational responses, along with attitudinal and behavioral reactions, to various beverages and snacks (such as oat milk and fruit smoothie with soy milk). Hence, this study included sensory appeal in the variables being studied.

Despite considerable previous work, the understanding of the motivators behind consumer inclinations towards plant‐based alternatives remains incomplete. The impact of consumers' food selection motives on their intentions for plant‐based products, though instrumental in predicting overall food intentions and behaviors, has not been fully explored (Hoek et al., 2011; Onwezen et al., 2021). It is widely agreed that health considerations are associated with an increased acceptance of plant‐based foods (Hoek et al., 2011; Marty et al., 2022; Moss et al., 2022; Siegrist & Hartmann, 2019). Likewise, environmental considerations boost the purchase intention for sustainable items (Onwezen et al., 2022).

To address these gaps, this study aimed to answer the following research questions:

1. What are the main factors influencing consumers' willingness and behavior to purchase plant‐based eggs?
2. How do personal attitudes, subjective norms, and perceived behavioral control outlined in the TPB affect the intention to purchase plant‐based eggs?
3. How do personal values related to health, the environment, and animal welfare, as captured by the VAB model, influence consumers' attitudes towards plant‐based eggs?
4. What role do dietary habits, specifically vegetarianism and religious beliefs, play in shaping consumers' intentions to purchase plant‐based eggs?
5. How can the TPB and the VAB model be adapted to better understand consumer purchasing intentions for plant‐based eggs?

It is hoped that the insights gained from this study will substantially aid product development and marketing strategies within the rapidly evolving plant‐based eggs sector. By providing a deeper understanding of key motivations and the relationship with consumers' perceived health‐related value of products, it aims to reconcile contradictory findings in prior research on consumer acceptance of sustainable food innovations and extend the established Theory model. This will hopefully deliver a nuanced understanding of consumers' environmentally sustainable food behaviors, highlighting the importance of their dietary values.

## 2 LITERATURE REVIEW AND HYPOTHESIS DEVELOPMENT

### 2.1 Theory of planned behavior

The TPB, introduced by Ajzen (1991), is a robust socio‐psychological model for elucidating human behavior. The TPB operates on the fundamental premise that attitudes, subjective norms, and perceived behavioral control guide a person's intentions, thus shaping their behavior. While Ajzen's original model identifies these three antecedents, it still advocates the inclusion of additional predictors as warranted by context (Ajzen, 1991). Given the unique attributes inherent to plant‐based eggs and the insights garnered from pertinent literature, this study expands TPB by integrating a sensory appeal dimension, as delineated below:

#### 2.1.1 Attitudes (AT)

Attitudes, enduring, and pervasive evaluations of certain entities are believed to predict behavioral inclinations (Eagly & Chaiken, 1993). As attested by Nardi et al. (2019), they represent beliefs about the anticipated outcomes of specific behaviors. Hence, the more favorable the attitude towards a behavior, the greater the likelihood of its execution. When considering plant‐based eggs, attitudes may encompass health benefits, nutritional value, and environmental conservation. Positive attitudes may augment consumers' intentions to engage.

#### 2.1.2 Subjective norms (SN)

Subjective norms denote societal pressures that influence behavior; the stronger these norms, the stronger the proclivity to act (Ajzen, 1991). As explained by Al‐Swidi et al. (2014) and Nardi et al. (2019), subjective norms are derived from formal (laws and guidelines) and informal powerful sources (peers, family, and media). Therefore, if consumers feel that those surrounding them approve of plant‐based eggs, their intent to consume them may increase.

#### 2.1.3 Perceived behavioral control (PBC)

Perceived behavioral control encapsulates an individual's perceived capacity to marshal resources and seize opportunities for specific actions (Ajzen, 1985). It comprises both internal control (personal skills, knowledge, and self‐discipline) and external control (time, finances, and opportunities) components (Ajzen, 1991). Increased resources and opportunities tend to amplify an individual's capacity to execute these actions (Chen et al., 2017). This element is especially crucial when behavior is partially beyond volitional control (Kamon‐ard, 2020). Shalender and Sharma (2021) pointed out in their study that when consumers believe that they can control these factors, their likelihood of engaging in green purchasing behavior increases. Hence, provided sufficient resources and capabilities, consumers may exhibit an increased readiness to engage with plant‐based eggs.

#### 2.1.4 Purchase intention (PI)

Behavioral intention refers to the magnitude and direction of an individual's propensity to engage in certain behaviors (Ajzen & Fishbein, 2005). As they often serve as proxies for actual behavior, measuring them can provide significant insights into actual tendencies (Ho et al., 2020; Lin & Roberts, 2020; Mailizar et al., 2021; Muangmee et al., 2021).

The extant literature demonstrates TPB's efficacy of TPB in predicting purchase intention. Canova et al. (2020) discovered through a TPB application that attitudes, subjective norms, and perceived behavioral control positively influenced organic food purchase intentions. Lim and An (2021) confirmed these factors as positive influencers of dietary supplement purchase intentions. Varah et al. (2021) confirmed that this theory is applicable to studies that focus on green products. Coşkun and Özbük (2020) demonstrated the theory's predictive ability regarding intentions to reduce food waste, with perceived behavioral control having the greatest impact on the willingness to reduce food waste.

Consequently, we can extrapolate that the intent to buy plant‐based poached eggs could depend on attitudes, subjective norms, and perceived behavioral control, prompting the following hypotheses:

*Attitudes have a significant and positive influence on consumers' intentions towards purchase plant‐based eggs*.

*Subjective norms have a significant and positive influence on consumers' intention towards purchase plant‐based eggs*.

*Perceived behavioral control has a significant and positive influence on consumers' intention towards purchase plant‐based eggs*.

Jham et al. (2023) emphasized that subjective norms strengthen the link between hedonic value in luxury co‐consumption and purchase intention. Ateş (2020) indicated that biospheric values, attitudes, perceived behavioral control, and environmental self‐identity directly influence personal norms, thereby impacting pro‐environmental behaviors. These findings underscore the argument that values indirectly influence purchase intention through intermediary variables. Therefore, this study proposes the following hypotheses:

*Attitude mediates the relationship between eating value and purchase intention*.

*Subjective norms mediate the relationship between eating values and purchase intention*.

*Perceived behavioral control mediates the relationship between eating values and purchase intention*.

### 2.2 Sensory appeal

Empirical evidence indicates that sensory attributes significantly influence consumer preferences and consumption intentions (Guedes et al., 2023; Imtiyaz et al., 2021), and are paramount in food selection (Hati et al., 2021; Mishyna et al., 2020). For instance, packaging color can influence consumers' perceptions and purchase intentions (Marozzo et al., 2020; Sucapane et al., 2021). Thus, the current study hypothesizes the following:

*Consumers' sensory appeal has a significant and positive influence on their intention towards purchase plant‐based eggs*.

### 2.3 Value‐attitude‐behavior (VAB) model

Homer and Kahle (1988) initially presented the Values, Attitudes and Behaviors (VAB) model, a conceptual framework corroborated by abundant empirical studies. These studies highlight the interconnectedness of personal values, attitudes, and subsequent behaviors. The application of structural equation modeling (SEM) in these investigations substantiates the direct or collateral influence of values on behaviors, predominantly via attitudes. For instance, Ma and Chang (2022) confirmed in their study of plant‐based meat that green values positively affect consumers' attitudes, which, in turn, influence their purchase intentions. Kim and Hall (2021) found that sustainable values and attitudes influence the behavior of Korean people towards sustainable crowdfunding.

With a nod to this model, Chang et al. (2021) integrated considerations such as “environmental concern,” “time pressure,” and “cooking habits” into their research on consumer behavior for precooked plant‐based foods (PPBFs), utilizing the VAB framework. Parallelly, Lee et al. (2021) incorporated concepts like “food neophobia” and “fear of food technology” as moderating variables into the VAB model to investigate consumer reactions to 3D‐printed food. Similarly, Govaerts and Olsen (2022) applied the VAB model to examine the effects of biospheric and hedonic values on consumers' propensity to purchase seaweed food. These findings reinforce the results of research by Ma and Chang (2022) and Kim et al. (2020) concerning environmentally friendly food preferences, which propound a reciprocal relationship between value, attitude, and behavior.

“Eating Values” pertain to an individual's value framework and convictions about eating habits and food selection (Nystrand & Olsen, 2020a). These values bifurcate into two principal aspects: utilitarian and hedonic. Utilitarian eating values encapsulate considerations of convenience, nutrition, and health, while hedonic eating values underscore the sensory gratification derived from taste and pleasure (Lusk & Briggeman, 2009). The innate utilitarian and hedonic values of most foods differ, and food products adept at appealing to both dimensions typically retain consumer loyalty (Steptoe et al., 1995). Contemporary research shows how these two types of values can steer consumers' actions and ensuing behavioral intentions, including purchase decisions and word‐of‐mouth publicity (Hur & Jang, 2015). Ghali (2020) applies utilitarian and hedonic values to explore their impact on Tunisian consumers' willingness to purchase and pay for organic olive oil. Chen et al. (2020) also investigated utilitarian and hedonic values concerning consumers' attitudes and purchase intentions towards delivery platform services.

Previous scholarly work chronicles explorations linking value dimensions with the TPB. For example, Nystrand and Olsen (2020b) used the TPB to gauge Norwegian consumers' inclination towards functional food consumption, considering food values as precursors of attitudes. Sadiq et al.'s (2021) study on organic food consumption underlines the material influence of food values on customer attitudes.

Informed by these insights, our current study predicts that, within the framework of landscape restaurants, values are apt to impart a substantially positive impact on attitude, subjective norms, and perceived behavioral control. The study proposes:

*Eating values have a significant and positive influence on consumers' attitudes towards purchasing plant‐poached eggs*.

*Eating values have a significant and positive influence on consumers' subjective norms towards purchasing plant‐poached eggs*.

*Eating values have a significant and positive influence on consumers' perceived behavioral control towards purchasing plant‐poached eggs*.

Shin et al. (2019) suggest that hedonic value plays a crucial role in shaping behavioral intentions, albeit utility value shows a non‐significant impact. Mason et al. (2023) find that consumer values have a significant positive impact on consumer behavior, with emotional values being the most influential predictor. Chattaraman et al. (2023) pointed out that the importance of healthy eating is increasing, and consumers' attitudes and purchase intentions towards healthier foods rise accordingly when they shop for themselves. Therefore, this study proposes the following hypotheses:

*Eating values have a significant and positive influence on consumers' intentions towards purchase plant‐based poached eggs*.

## 3 MATERIALS AND METHODS

### 3.1 Research framework

**FIGURE 1:**
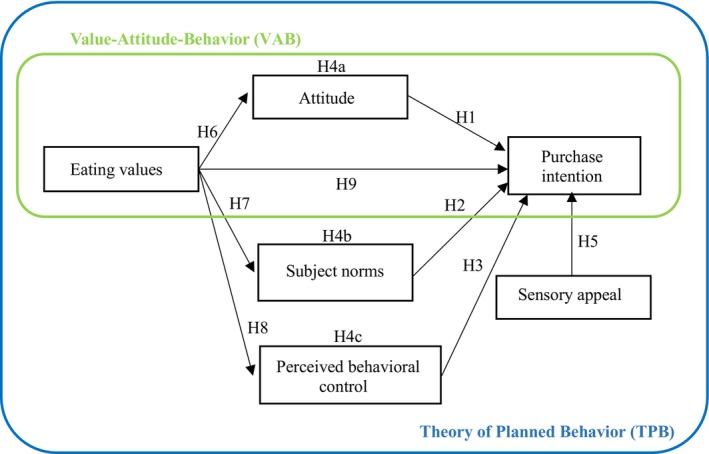
Research framework.

Drawing upon the examined literature, this research broadens the scope of the TPB framework. The original TPB constituents – specifically attitude, subjective norms, perceived behavioral control, and purchase intention – maintain their foundational roles within this expanded paradigm. Moreover, this study integrated two additional dimensions: eating value and sensory appeal. Driven by the literature, these new elements further enhance the applicability of the TPB framework. A revised and comprehensive research framework is presented in Figure 1.

### 3.2 Questionnaire design

The questionnaire design was compartmentalized into seven distinct sections. Section one, focusing on eating values, draws upon the studies of Pérez‐Villarreal et al. (2020) and Nystrand and Olsen (2020a), comprising eight items. Section two constitutes a revised attitude scale, adapting the work of Qi and Ploeger (2023) and encompassing three items. Section three pertains to subjective norms, inspired by Liu et al. (2022), using three items. The fourth division explores perceived behavioral control, revised from the studies of Sabbagh et al. (2023) and Qi and Ploeger (2021), comprising five items. Purchase intentions form the basis of the fifth part, adapted from Zheng et al. (2022) and incorporating five items. Section six addresses sensory appeal, inspired by Trinh et al. (2023), and consists of four items. The seventh section obtained demographic information from the participants, including gender, age, educational background, income, religious beliefs, and food culture. With the exception of demographic data, all elements of the questionnaire employed a seven‐point Likert scale, where 1 signified “strongly disagree” and 7 represented “strongly agree.”

Following the completion of the questionnaire, a validity review was conducted by experts to curtail any potential misunderstandings arising from ambiguous semantics or incomplete descriptions. This process engages the expertise of educational scholars and established professionals in the food industry, among others, totaling nine, each with a minimum of a decade of work experience. After obtaining consent, the questionnaire was forwarded to the experts for a critical evaluation of each item's accuracy, suitability, and phraseology. Upon collation of expert opinions, suggestions were integrated to refine the semantic value and to modify, add, or delete questionnaire items, thus leading to a pilot questionnaire.

For the pilot phase, 105 questionnaires were distributed, returning 92 valid responses, all of which were subjected to item and reliability analyses to validate the questionnaire's restructured components. Upon collection of the formal questionnaire, responses failing reverse question tests or consistently similar entries, as well as incomplete ones, were deemed invalid. The internal consistency reliability of each construct, represented by Cronbach's alpha, ranged from 0.828 to 0.953, demonstrating the reliability of the questionnaire design.

### 3.3 Sample and data collection

In the evolving digital age, the convenience and broad reach of the internet and social media have significantly shifted social science research methodologies. Researchers have shifted from traditional paper‐based questionnaires to digital platforms, which offer convenient and eco‐friendly approaches. According to Sammut et al. (2021), online questionnaires, despite having lower response rates, can enhance participation through preemptive emails or by designing compact, 10‐min questionnaires. They also offer improvements compared to their paper‐based counterparts by ensuring data integrity, preserving resources, and providing more comprehensive responses.

Convenience sampling was used in this study. This nonprobability sampling technique was chosen because of its efficiency in quickly and cost‐effectively collecting data from a readily available pool of respondents. The questionnaires were distributed through various widely used platforms including Facebook, Instagram, and Line groups and communities. This multiplatform strategy aimed to maximize the diversity and reach of our sample, capturing a wide range of demographics.

This study was conducted rigorously within the geographical confines of Taiwan. This locale was selected based on Taiwan's dynamic consumer market and its significant adoption of digital marketing strategies, particularly in relation to sustainable food purchasing behaviors. Taiwan is characterized by a high Internet penetration rate and growing public awareness of sustainable consumption practices, making it an academically rich environment for investigating the impact of plant‐based products.

In alignment with ethical research guidelines, the survey clearly stated its purpose and assured respondents of their anonymity on the questionnaire's initial page. This approach was intended to foster a sense of security and to encourage participants to share their responses without concerns about privacy breaches.

For hypothesis validation and to achieve the objectives of this study, data analysis was performed using SEM. According to Wu (2009), the optimal sample size for such modeling lies within the ratio of 10:1 to 15:1 concerning the number of questions. Considering that our questionnaire consisted of 23 questions, the ideal sample size ranged from 230 to 345.

The questionnaire was distributed between July and August 2023 and 279 responses were received. After discarding 40 invalid responses, 239 valid questionnaires were retained for the analysis. Table 1 presents the participants' demographic details.

**TABLE 1:** Demographic analysis.

*N* = 239	Item	Population	Percentage (%)
Gender	Male	88	36.8
	Female	151	63.2
Age	20 years and below	14	5.9
	21–30 years	65	27.2
	31–40 years	43	18.0
	41–50 years	63	26.4
	51–60 years	49	20.5
	61 years and above	5	2.1
Level of education	Middle school or below	10	4.2
	High school/vocational	49	20.5
	College/university	134	56.1
	Master's or above	46	19.2
Monthly personal income	Less than NTD$20,000 (660 USD) (inclusive)	46	19.2
	NTD$20,001–40,000 (660–1320 USD)	71	29.7
	NTD$40,001–60,000 (1320–1980 USD)	54	22.6
	NTD$60,001–80,000 (1980–2640 USD)	44	18.4
	Above NTD$80,001 (2640 USD)	24	10.0
Dietary habits	Omnivorous	227	95.0
	Vegan	0	0
	Ovo‐vegetarian	0	0
	Lacto‐vegetarian	0	0
	Lacto‐ovo vegetarian	7	2.9
	Bodhi vegetarian	5	2.1
Religious belief	Non‐religious	96	40.2
	Buddhism and Taoism	136	56.9
	Christianity	3	1.3
	Catholicism	0	0
	Other	4	1.7

Abbreviation: NTD, New Taiwan dollar (1 NTD = 0.033 USD).

### 3.4 Methods of data analysis

Adopting a quantitative research approach, this study employed questionnaire surveys for data gathering and utilized IBM SPSS Statistics 27 and AMOS 28 for intricate data analysis. The statistical analysis tools implemented in this study encompass an array of techniques. These include descriptive statistics, frequency distribution tables, percentages, means, and standard deviations to expound upon our core numerical data. To ensure that the collected data met the standards of consistency and accuracy, their reliability and validity were scrutinized.

Furthermore, to comprehend complex interdependencies, the Maximum Likelihood Estimation (MLE) method for structural equations was employed. This elucidated the causal relationships between the variables and evaluated the overall fit of the underlying models. This extensive methodological process facilitated the validation of the hypotheses articulated in this research.

## 4 ANALYSIS AND RESULTS

### 4.1 Measurement model: Reliability and validity

In this study, a two‐step approach to analysis was adopted. The first stage comprised Confirmatory Factor Analysis (CFA), while the second stage was dedicated to the overall model's fitting. The CFA, acting as an integral part of SEM, scrutinizes the correlation between observed variables and latent factors to confirm whether the latter is adequately represented by the former. By evaluating psychological measurement, construct validity, testing method effectiveness, and model group invariance, CFA holds value in contexts where predeveloped questionnaires are used, requiring validation for their appropriateness for the study population.

This study comprises six sub‐dimensions: “Eating Values,” “Attitude,” “Subjective Norm,” “Perceived Behavioral Control,” “Sensory Attraction,” and “purchase Intention.” Each sub‐facet underwent CFA; initially, items with factor loadings below 0.4 were extracted based on the results. Sequential CFAs were conducted to assess the Root Mean Square Error of Approximation (RMSEA) of the sub‐facet, suggesting unfit standards when the figure surpassed 0.08. Model revisions were performed iteratively using the MI value of the modification index until each facet's RMSEA dropped below 0.08 or became a saturated model. For instance, “Eating Values” was reduced from six to five items, and “Perceived Behavioral Control” slimmed down from four to three items.

After the scale's sub‐dimensions were set, the Composite Reliability (CR) and convergent validity for each scale dimension were promptly tested. The CR value, ranging from 0 to 1, measures reliability across variables. A higher CR value signifies augmented “true variation to total variation,” that is, enhanced internal consistency. Previous research by Fornell and Larcker (1981) suggests that the latent variables' CR value should surpass 0.60, while the Average Variance Extracted (AVE), representing the convergent validity of latent variables, should ideally exceed 0.50.

For this study, each dimension of CR ranged from 0.898 to 0.971, suggesting commendable internal consistency. The AVE spanned between 0.746 and 0.917, exceeding the recommended value of 0.50, thereby indicating high convergent validity. Details of the factor loadings, CR values, and AVE values are summarized in Table 2, revealing that each aspect of the questionnaire adequately met the convergent validity requirements, thereby guaranteeing the intrinsic quality of the measurement model.

**TABLE 2:** Results related to factor loading, reliability, and validity.

Variables/items	Mean	Standard deviation	Standardized factor loading	AVE	CR
Eating values (EV)	4.732	1.292		0.756	0.939
1. I believe the convenience of plant‐based poached eggs provides a pleasant experience	4.540	1.451	0.842***		
2. I believe the environmental impact of plant‐based poached eggs is exciting	5.010	1.513	0.848***		
3. I am satisfied with the nutritional value of plant‐based poached eggs	4.620	1.427	0.888***		
4. I believe the environmental impact of plant‐based poached eggs is beneficial and necessary	4.860	1.515	0.894***		
5. I believe plant‐based poached eggs can help prevent health problems	4.630	1.522	0.874***		
Attitude (AT)	4.324	1.439		0.917	0.971
1. I believe purchasing plant‐based poached eggs is a wise choice	4.330	1.440	0.959***		
2. I find the act of purchasing plant‐based poached eggs pleasurable	4.300	1.487	0.972***		
3. I believe purchasing plant‐based poached eggs is of considerable importance	4.330	1.587	0.941***		
Subject norms (SN)	3.837	1.399		0.864	0.950
1. My family and friends think I should choose to eat plant‐based poached eggs	3.680	1.574	0.936***		
2. My superiors and colleagues support me when I buy plant‐based poached eggs	4.000	1.395	0.905***		
3. Most people who matter to me believe I should purchase plant‐based poached eggs	3.820	1.546	0.947***		
Perceived behavioral control (PBC)	3.593	1.568		0.746	0.898
1. I clearly know where to purchase plant‐based poached eggs	3.050	1.845	0.903***		
2. I have knowledge on how to eat plant‐based poached eggs appropriately	3.530	1.824	0.913***		
3. If I have the will, purchasing plant‐based poached eggs is easy	4.200	1.784	0.768***		
Purchase intention (PI)	4.145	1.549		0.810	0.955
1. I have a strong intention to deepen my understanding of plant‐based poached eggs	4.300	1.681	0.903***		
2. I would consider purchasing plant‐based poached eggs	4.470	1.707	0.937***		
3. I would recommend plant‐based poached eggs to others	4.140	1.640	0.943***		
4. I am willing to pay a higher price to purchase plant‐based poached eggs	3.470	1.784	0.830***		
5. If I have enough time, energy, and financial resources, I would purchase plant‐based poached eggs	4.340	1.812	0.883***		
Sensory appeal (SA)	3.923	1.361		0.843	0.956
1. I believe the taste of plant‐based poached eggs is pleasant	3.780	1.459	0.933***		
2. I think the appearance of plant‐based poached eggs is exquisite and visually appealing	4.140	1.556	0.886***		
3. I believe the aroma of plant‐based poached eggs is pleasing	3.820	1.423	0.943***		
4. I find the taste of plant‐based poached eggs delicious and appealing	3.950	1.493	0.910***		

Abbreviations: AVE, average variance extracted; CR, composite reliability.

*** *p* < .001.

Discriminant validity analysis is the final step in SEM, which compares two distinct concepts through a correlational analysis. A low correlation suggests discriminant validity between the concepts. Hair et al. (2019) suggested that the correlation coefficient between two different concepts should be less than the square root of AVE. Table 3 compares all construct correlation coefficients and the square roots of the AVEs in this study, showing that each construct's AVE square root surpasses the correlation coefficients, which aligns with Hair et al.'s (2019) standard and confirms discriminant validity among the constructs. As such, this study's measurement model demonstrated satisfactory internal and external qualities.

**TABLE 3:** Discriminant validity test.

Variables	EV	AT	SN	PBC	PI	SA
EV	**0.869**					
AT	0.811**	**0.957**				
SN	0.631**	0.791**	**0.930**			
PBC	0.412**	0.481**	0.536**	**0.864**		
PI	0.723**	0.787**	0.727**	0.525**	**0.900**	
SA	0.634**	0.708**	0.747**	0.624**	0.775**	**0.918**

*Note*: The value in bold font is the square root of the AVE; the non‐diagonal numbers represent the correlation coefficients of each dimension.

Abbreviations: AT, attitude; EV, eating values; PBC, perceived behavioral control; PI, purchase intention; SA, sensory appeal; SN, subjective norms.

** *p* < .01.

### 4.2 Model fit test

The present study utilizes the Maximum Likelihood (ML) method to formulate a structural model that scrutinizes the theoretical relationships proposed by the model under study. The following illustrates the outcomes: × (Ajzen, 1985)/*df* equates to 2.861, the goodness‐of‐fit index (GFI) is logged at 0.806, the root mean square error of approximation (RMSEA) is estimated at 0.088, the Tucker‐Lewis Index (TLI) is 0.921, the incremental fit index (IFI) is 0.933, and the comparative fit index (CFI) is 0.933. These indices fulfilled the standard requirements.

Bagozzi and Yi (1988) articulated that a GFI exceeding 0.90 is rigidly satisfactory, whereas a value surpassing 0.80 is considered acceptable. Considering that the majority of the fit indices in this study either attained or surpassed these standards, it can be inferred that the model exhibits a robust correlation with the data, signifying its optimal alignment with the expected outcomes.

### 4.3 Overall model path analysis

The research hypotheses presented in Table 4 were validated in this study, drawing upon the Extended TPB and the VAB Model.

**TABLE 4:** Results of the path analysis and confirmation of hypotheses.

Hypothesized paths	Unstandardized coefficient	SE	CR	*p*	Standardized coefficients	*β*	Verification results
H1:AT → PI	0.224	0.139	1.613	***	0.205	0.787	Supported
H2:SN → PI	0.045	0.079	0.572	***	0.044	0.727	Supported
H3:PBC → PI	−0.034	0.047	−0.723	***	−0.037	0.525	Unsupported
H5:SA → PI	0.428	0.080	5.350	***	0.383	0.775	Supported
H6:EV → AT	1.105	0.073	15.209	***	0.921	0.811	Supported
H7:EV → SN	1.004	0.084	11.988	***	0.789	0.631	Supported
H8:EV → PBC	0.734	0.103	7.153	***	0.510	0.412	Supported
H9:EV → PI	0.438	0.177	2.474	***	0.334	0.723	Supported

Abbreviations: AT, attitude; EV, eating values; PBC, perceived behavioral control; PI, purchase intention; SA, sensory appeal; SN, subjective norms.

*** *p* < .001.

#### 4.3.1 Insights from the tests conducted on various hypotheses of the extended theory of planned behavior

Consumer attitudes significantly positively impact the behavioral intention to purchase plant‐based poached eggs (*β* = 0.787, *t*‐value = 19.611; *p* < .001), thereby confirming H1. The subjective norms of consumers significantly positively influence the behavioral intention to buy plant‐based poached eggs (*β* = 0.727, *t*‐value = 16.287; *p* < .001); hence, H2 is accepted. However, the perceived behavioral control of consumers negatively and significantly impacts the behavioral intention to purchase plant‐based poached eggs (*β* = 0.525, *t*‐value = 9.500; *p* < .001), thus rejecting H3. Among the expanded facets of the extended Theory, the sensory appeal of consumers significantly positively influences the behavioral intention to purchase plant‐based poached eggs (*β* = 0.775, *t*‐value = 18.906; *p* < .001), thus substantiating H5.

The test results showed that the alignment of consumer attitudes and subjective norms positively influenced behavioral intention to purchase plant‐based poached eggs, consistent with Ajzen's (1991) TPB. However, perceived behavioral control significantly negatively affects purchasing intent, aligning with Yzer (2012), who stated that perceived behavioral control does not always directly influence behavior, as environmental factors may serve as hindrances. In validating extended facets in the extended Theory, the elevated relevance of sensory attractiveness in influencing the behavioral intention to buy plant‐based poached eggs was noticeable.

#### 4.3.2 Results related to the hypotheses testing different paths in the value‐attitude‐behavior model

Consumers' eating values for plant‐based poached eggs significantly positively instigates their attitude towards buying plant‐based poached eggs (*β* = 0.811, *t*‐value = 21.350; *p* < .001), thereby verifying H6. Furthermore, consumers' eating values for plant‐based poached eggs had a significant positive influence on the subjective norms for buying plant‐based poached eggs (*β* = 0.631, *t*‐value = 12.516; *p* < .001), thus verifying H7. Additionally, this dietary value significantly and positively affected perceived behavioral control (*β* = 0.412, *t*‐value = 6.690; *p* < .001), thereby confirming H8. Finally, eating values significantly and positively induced the behavioral intention to buy plant‐based poached eggs (*β* = 0.723, *t*‐value = 16.097; *p* < .001), ultimately verifying H9.

Building on the VAB model, the research results show that consumers' eating values for plant‐based products considerably and positively impact attitudes, subjective norms, perceived behavioral control, and sensory appeal. The original VAB model encapsulated a direct sequence from value to attitude, culminating in behavior. The results of the hypothesis testing in this context suggest that eating values not only significantly influence attitudes but also affect other components of the TPB, such as subjective norms, perceived behavioral control, and the expanded facet of sensory appeal. This study enhances the VAB model by broadening the mediating factors between value and behavior.

### 4.4 Mediation effect test

The mediation effects in this study were tested according to Baron and Kenny's (1986) criteria, with the illustrative data provided in Table 5. The results indicated that a mediation effect was present in various sequences.

**TABLE 5:** Mediating effect regression analysis.

	AT	PI		
	Model 1	Model 2	Model 3	Model 4
EV	0.811***	0.723***		0.248***
AT			0.787***	0.586***
*R* ^2^	.658	.522	.619	.640
Agj *R* ^2^	.656	.520	.617	.637
*F*	455.811***	259.128***	384.574***	209.485***
Degrees of freedom	(1237)	(1237)	(1237)	(2236)

	SN	PI		
	Model 1	Model 2	Model 3	Model 4
EV	0.631***	0.723***		0.439***
SN			0.727***	0.450***
*R* ^2^	.398	.522	.528	.644
Agj *R* ^2^	.395	.520	.526	.641
F	156.658***	259.128***	265.259***	213.576***
Degrees of Freedom	(1237)	(1237)	(1237)	(2236)

	PBC	PI		
	Model 1	Model 2	Model 3	Model 4
EV	0.412***	0.723***		0.610***
PBC			0.525***	0.274***
*R* ^2^	.170	.522	.276	.585
Agj *R* ^2^	.166	.520	.273	.581
F	48.445***	259.128***	90.247***	166.056***
Degrees of Freedom	(1237)	(1237)	(1237)	(2236)

*Note*: The values in table are standardized regression coefficients (*β*).

*** *p* < .001.

For the mediation sequence Eating Values (EV) → Attitudes (AT) → Purchase Intentions (PI), a significant impact was observed for EV on both AT (*β* = 0.811, *p* < .001) and PI (*β* = 0.723, *p* < .001), and from AT on PI (*β* = 0.787, *p* < .001). When the simultaneous impacts of EV and AT on PI are considered, the predictive power *β* of EV decreases from the original value of 0.723 to 0.248, and the explanatory power *R* ^2^ increases from the initial .522 to .640. Thus, the mediation effect is confirmed.

Similarly, in the EV → Subjective Norms (SN) → PI sequence, EV significantly influenced SN (*β* = 0.631, *p* < .001), and SN significantly affected PI (*β* = 0.727, *p* < .001). When the concomitant impacts of EV and SN on PI were considered, the predictive power *β* of EV decreased from the original value of 0.723 to 0.439, and the explanatory power *R* ^2^ increased from the initial .522 to .644. Thus, a mediating effect was established.

Lastly, in the EV → Perceived Behavioral Control (PBC) → PI sequence, EV significantly impacts PBC (*β* = 0.412, *p* < .001), which, in turn, significantly affects PI (*β* = 0.525, *p* < .001). Considering the impact of EV and PBC on PI concurrently, the predictive power *β* of EV was reduced from the original 0.723 to 0.610, and the explanatory power *R* ^2^ increased from the initial .522 to .585. Thus, the mediation effect was confirmed.

## 5 DISCUSSION

### 5.1 Theoretical implications

This research utilizes the TPB and VAB models to delve into consumer intention towards the purchase of plant‐based poached eggs. The TPB‐based findings revealed that attitude and subjective norms markedly influence the intention to buy plant‐based poached eggs, corroborating previous studies (Lim & An, 2021; Setiawati et al., 2018; Shin et al., 2020). Conversely, perceived behavioral control negatively influences this intention, aligning with Kim, Kim, and Han's (2021) research, which noted a lack of significant impact of perceived behavioral control on behavioral intentions. This study also reveals the increased intention to consider and purchase plant‐based poached eggs based on consumer attitudes and approval from their social circles. However, an increase in pricing dampens purchase intentions. Moreover, the study broadened the scope of TPB by considering sensory appeal, which significantly influences purchasing intention among inexperienced buyers, consistent with prior research (Marozzo et al., 2020; Sucapane et al., 2021).

In recent years, increasing consumer desire for environmental protection and personal health has led to an increase in the number of vegetarians. However, the results indicated that vegetarians' behavioral intention to purchase plant‐based eggs did not increase accordingly. Kim et al. (2022) noted that vegetarians prioritize ethical concerns, health, convenience, and price, while omnivores prioritize sensory appeal and weight control, yet both groups hold positive attitudes towards plant‐based foods. This finding aligns with the results of the present study, suggesting that support for purchasing plant‐based eggs may exist, regardless of whether individuals are vegetarians. No clear correlations were observed regarding religious beliefs. However, Hasan et al. (2024) found a positive relationship between consumers' religious beliefs and willingness to purchase organic foods, which contradicts the findings of this study. This discrepancy may be due to differences in the sample characteristics. Hasan et al.'s study often focused on Islam, whereas this study's sample predominantly consisted of Buddhists. Different religious beliefs entail distinct dietary norms and cultural influences that can affect dietary choices and consumption behaviors. Therefore, the impact of various religious beliefs on the intention to purchase sustainable food is worthy of further research.

Additionally, this study integrates the value aspect of TPB by considering consumers' perceived eating values of plant‐based poached eggs, including aspects such as convenience, environmental friendliness, and nutritional value. This integration resulted in a research framework reminiscent of VAB, but with enhanced novelty through the addition of two more mediators, subjective norms and perceived behavioral control, between value and purchase intention.

Previous research utilizing the VAB model has acknowledged that value directly or indirectly influences consumer behavioral intentions (Chang et al., 2020; Latif et al., 2019; Lee et al., 2019). Compared to the original VAB model that incorporated attitude as the sole mediator, this study broadened the mediation model using TPB, attributing the influence on purchase intention to attitude along with subjective norms, perceived behavioral control, and eating values. This research also underscores the positive influence of perceived value on the formation of attitudes, which resonates with the findings of Fiandari et al.'s (2019) study on repeated fish consumption attitudes. Research conducted by Kim, Kim, and Hwang (2021), focusing on the intent to use drone delivery services, found attitudes, subjective norms, and perceived behavioral control as significant determinants of behavioral intentions. This study broadens this model by investigating consumers' behavioral intentions to purchase plant‐based poached eggs. The findings revealed that eating values indirectly and positively affect purchase intention through attitudes and subjective norms, while being negatively influenced indirectly through perceived behavioral control. Thus, the research substantiates the extended VAB model in the context of consumer intentions to purchase plant‐based poached eggs.

### 5.2 Management implications

The findings of this study offer insights into the mediating variables between eating values and purchase intention. Influencing consumers' attitudes towards plant‐based poached eggs could enhance their purchase intentions. Businesses may employ mass media and online marketing strategies to shape the perceptions of key customer reference groups and influence subjective norms. In the present study, an insufficient understanding of plant‐based poached eggs negatively impacted buying intentions. Consequently, making information easily accessible and comprehensible can improve perceived behavioral control, thereby enhancing buying intentions.

Producers should also focus on elevating the inherent value of the product to ensure consumers recognize their worth, consequently leading to higher purchase intentions. In this context, targeted marketing strategies such as capitalizing on social media exposure can prove beneficial. As consumers share their plant‐based egg‐eating experiences, they contribute to the information pool about these products, enhancing the perceived value of plant‐based eggs and subsequently increasing their attitudes, subjective norms, perceived behavioral controls, and sensory attractions. Therefore, the intention to buy plant‐based poached eggs is amplified.

## 6 CONCLUSIONS

### 6.1 Research conclusions

It has been found that perception of eating values impacts purchase intention through three distinct facets: attitudes, subjective norms, and perceived behavioral control. Specifically, for businesses involved in the production of plant‐based poached eggs, augmenting consumers' recognition and appreciation of dietary values can positively influence their purchase intentions, provided that attitudes, perceived behavioral control, and subjective norms are deemed significant. This means that if consumers recognize and associate positive values with eating plant‐based eggs, including a positive attitude, garnering support from social circles, having the financial capacity and time to access information about these products, and finding them sensorially pleasing, are likely to escalate their intent to purchase plant‐based eggs.

This study provides evidence of a significant correlation between eating values and purchase intention. It was discovered that attitudes, subjective norms, and perceived behavioral control served as significant mediatory variables between eating values and the intent to purchase plant‐based poached eggs. This indicates that while eating values directly shapes consumers' intent to buy plant‐based poached eggs, these values also exert an indirect influence on purchasing decisions, which is mediated through one or multiple intermediary variables.

### 6.2 Research limitations and future research directions

In the contemporary context, significant concerns, such as protein deficiencies, sustainability, food safety, and health benefits, have become globally salient. Nonetheless, consumer knowledge of plant‐based eggs remains relatively scant. When informed about the environmental sustainability and health benefits of such alternatives, consumers are willing to learn more and contemplate purchasing. Consequently, environmental consciousness and health considerations have emerged as the primary factors driving consumer acceptance of plant‐based eggs.

Future research aimed at augmenting the understanding of consumers' purchase intention towards plant‐based foods is recommended to increase the sample diversity of respondents from various geographical regions. It would be beneficial to consider variables, such as environmental sustainability and health concerns, in the research framework to discern their impact on consumers' decisions. Additionally, more extensive research on consumers' WTP can provide valuable insights into this domain. Thus, expanding the scope and depth of inquiry may enrich existing literature in this area.

## AUTHOR CONTRIBUTIONS

**Han‐Shen Chen:** Conceptualization (lead); data curation (equal); methodology (lead); validation (equal); visualization (equal); writing – original draft (lead); writing – review and editing (lead). **Ching‐Tzu Chao:** Data curation (equal); investigation (equal); methodology (supporting); validation (equal); visualization (equal); writing – original draft (supporting). **I‐Kai Lin:** Data curation (equal); investigation (equal); methodology (supporting); validation (equal); visualization (equal).

## FUNDING INFORMATION

This study received no external funding.

## CONFLICT OF INTEREST STATEMENT

The authors declare no conflicts of interest.

## Data Availability

The data supporting the findings of this study are available from the corresponding author, H.‐S.C., upon reasonable request.
